# Bone marrow edema-like signal after cartilage repair does not affect outcomes in a five-year follow-up

**DOI:** 10.1007/s00330-024-11078-8

**Published:** 2024-09-16

**Authors:** Felix R. M. Koenig, Marcus Raudner, Vladimir Juras, Pavol Szomolanyi, Veronika Vetchy, Jakob Kittinger, Ehsan Safai Zadeh, Martin L. Watzenböck, Siegfried Trattnig

**Affiliations:** 1 Department of Biomedical Imaging and Image-Guided Therapy, High-Field MR Centre, Medical University of Vienna, Vienna, Austria; 2 Department of Biomedical Imaging and Image-Guided Therapy, Medical University of Vienna, Vienna, Austria; 3 Institute of Measurement Science, Slovak Academy of Sciences, Bratislava, Slovakia; 4 CD Laboratory for Clinical Molecular MR Imaging, Vienna, Austria; 5 Austrian Cluster for Tissue Regeneration, Vienna, Austria; 6 Institute for Clinical Molecular MRI in the Musculoskeletal System, Karl Landsteiner Society, Vienna, Austria

**Keywords:** Knee joint, Articular cartilage, Subchondral arthroplasty, Magnetic resonance imaging, Follow-up studies

## Abstract

**Objectives:**

Bone marrow edema-like signal (BMELS) after cartilage repair is common, but its clinical significance remains uncertain. This study aimed to investigate the clinical and structural significance of BMELS following microfracturing (MFX) and matrix-induced autologous chondrocyte implantation (MACI).

**Methods:**

In this multicenter study, MRI examinations were performed over a period of 5 years after cartilage repair surgery (MFX *n* = 17; MACI *n* = 28) in 45 patients. Morphological assessments, including the MOCART 2.0 (magnetic resonance observation of cartilage repair tissue), quantitative imaging biomarkers (QIB) with T2 mapping of the repair tissue, and, specifically, assessment of the presence and size of BMELS, were conducted along with patient-reported outcome measures, such as the Knee injury and Osteoarthritis Outcome Score (KOOS) and the International Knee Documentation Committee (IKDC). BMELS structural and clinical assessments were obtained after 3 months, 12 months, and 60 months. Statistical analysis included the Mann–Whitney *U*-test, Wilcoxon rank test, Shapiro–Wilk test, and simulation-based power analysis.

**Results:**

BMELS were a common finding 60 months after cartilage repair. The size of BMELS differed significantly only between MACI and MFX patients after 3 months, with larger BMELS occurring in the MFX group. There were no significant differences in patients with or without BMELS regarding the T2 ratio of the treated area, the MOCART 2.0, or clinical scores.

**Conclusion:**

BMELS frequently appeared after cartilage repair procedures. We could show that the postoperative size and change in the size of BMELS after MACI and MFX did not affect clinical scores, morphological MRI results, or biochemical properties of the treated area after 60 months.

**Key Points:**

***Question*** *What is the clinical significance of bone marrow edema-like signal (BMELS) appearance after matrix-induced autologous chondrocyte implantation (MACI) or microfracture (MFX)*?

***Finding*** *There were no significant differences in patients with or without BMELS regarding the T2 ratio of the treated area, the MOCART 2.0, or clinical scores*.

***Clinical relevance*** *BMELS frequently appeared after cartilage repair, the appearance or the size dynamic after MACI and MFX did not affect clinical scores, morphological MRI results, or biochemical properties after 60 months*.

## Introduction

The successful treatment of cartilage lesions remains a challenge in daily clinical practice. The two most performed procedures to address this challenge are microfracturing (MFX) and matrix-induced autologous chondrocyte implantation (MACI). One common finding after cartilage repair procedures is bone marrow edema-like signal (BMELS) abnormalities [1].

However, the role and importance of subchondral BMELS remain uncertain and have been the subject of extensive discussion. Some studies report that these non-cystic subchondral hyperintensities resolve in the medium term, as long-term persistence may be associated with osteophyte formation and subchondral cyst formation, resulting in poorer outcomes [2]. Others claim that, especially during the early postoperative phase, the occurrence of BMELS is regarded as an inherent component of the physiological healing process, and, conversely, the lack of BMELS in the short postoperative course is considered a negative prognostic factor [3].

The aim of this study was to evaluate the appearance and course of BMELS over multiple postoperative follow-ups up to 60 months in 45 patients after cartilage repair of the knee using MFX or MACI. We correlated these findings with clinical and morphological scores and evaluated whether the treated cartilage area exhibits compositional changes associated with incidence or change in the size of BMELS, which can lead to OA in the long term.

## Methods

### Study design and inclusion criteria

This retrospective data analysis included patients from a multicenter, prospective, randomized controlled, and open-label study. The participants were provided with comprehensive information about their involvement in this multicenter, prospective, randomized controlled, and open-label study. They subsequently provided written consent after being fully informed. The research was conducted in compliance with the principles outlined in the Declaration of Helsinki and received approval from the ethical committees and regulatory authorities at each site involved in the study.

Inclusion criteria are summarized in the Supplementary Material.

The present retrospective analysis comprised 45 patients. These patients had a morphological MR imaging protocol and a T2 mapping sequence and underwent MRI exams at all follow-up time points (3 months, 12 months, and 60 months post-cartilage repair surgery). Of the whole sample, 28 individuals (62.2%) belonged to the MACI group, whereas 17 individuals (37.5%) were categorized under the MFX group.

### MR examination

Details of the MR examination are in Table 1 and the electronic Supplementary Material.

**Table 1:** Parameters of the MRI protocol used in this multicenter trial

Parameter/sequence	T2 mapping	TSE PD	TSE T2w	SE T1w
Orientation plane	Sagittal	Coronal	Sagittal	Sagittal
Slice thickness (mm)	3	3	2	2
Number of slices	17	25	29	29
Slice spacing (mm)	3.3	3.3	2.2	2.2
Repetition time (ms)	2000	3080	3310	700
Echo time (ms)	12.5; 25; 37.5; 50; 62.5; 75; 87.5; 100	28	85	12
Averages	1	2	3	1
Flip angle (°)	90, 180	180	180	90
Acquisition matrix	320 × 256	448 × 403	381 × 448	448 × 381
Image matrix	320 × 320	896 × 896	448 × 448	448 × 448
Pixel bandwidth (Hz/pix)	401	180	140	120
Field of view (cm)	16 × 16	16 × 16	16 × 16	16 × 16
Total acquisition time (min:s)	10:36	04:46	03:55	03:53

### Image analysis

**Fig. 1:**
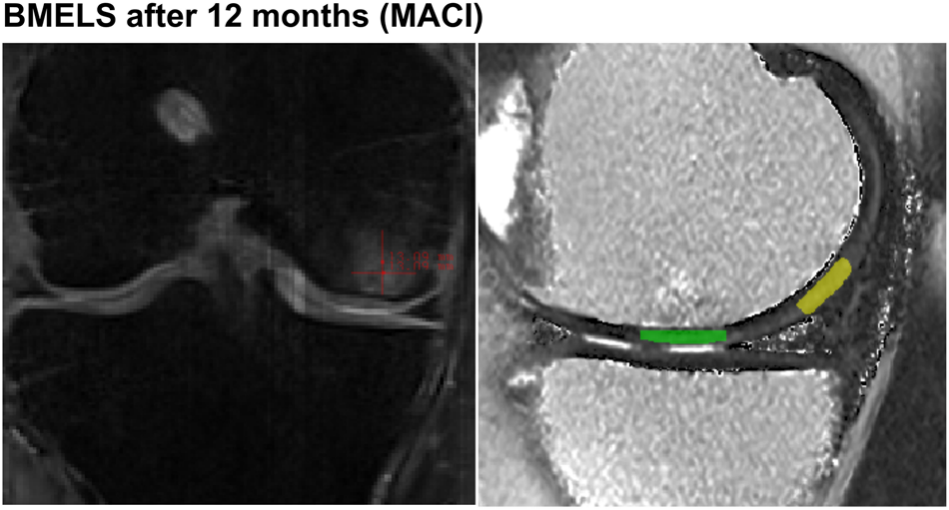
New appearance of BMELS after 12 months after MACI. This specific subchondral edema was getting smaller after 60 months. The size calculation is shown in red. The T2 ratio was calculated between the T2 values of the transplant area (green) and the reference cartilage of the femur (yellow). The left image depicts a measurement on a PD fs sequence and the right image shows the T2 mapping. Both images were taken from the image analysis tool used (ITK-SNAP)

The T2 values were assessed via ITK SNAP by the authors M.R. (senior radiologist with over eight years of experience in MSK imaging) and F.R.M.K. (resident radiologist with 3 years of experience in MSK Imaging). A minimum of three regions of interest (ROIs) were delineated within the cartilage repair tissue located in the indicated sites of cartilage repair. Cartilage repair sites were evaluated on three consecutive sagittal sections in all instances. To achieve an individual reference for the T2 values of each patient, we defined femoral cartilage that appeared morphologically homogenous and was not directly close to the repair tissue. Subsequently, three ROIs were drawn in at least three consecutive sagittal sections, as illustrated in Fig. 1.

The mean T2 values of the repaired tissue were divided by the mean T2 values of the morphologically inconspicuous femur cartilage. The same orientation relative to B0 as the graft tissue was chosen, to exclude the magic angle effect. The global T2 ratio that was established in this manner was utilized for the purpose of conducting the statistical analysis. The reproducibility of T2 mapping has already been shown in several other studies [4, 5].

BMELS were assessed on fat-suppressed proton density (PD) MRI sequences in the coronal plane by two authors (M.R. and F.R.M.K.) individually. Perfect interrater agreement (kappa = 1) was found for the presence of BMELS at 3 months, 12 months, and 60 months and for specific types of BMELS. Intraclass correlation was excellent (ICC > 0.99) for the size of BEMLS at 3 months, 12 months, and 60 months. The dimensions of the lesion were assessed longitudinally and transversely at 3 months, 12 months, and 60 months following cartilage repair, measuring the lesion at its largest dimension (Fig. 1). The extent of any BMELS area was calculated for each lesion (size (longitudinal) × size (transversal)). BMELS was classified into two categories, as described in the magnetic resonance observation of cartilage repair tissue scoring system (MOCART) 2.0 [6]: minor, where the maximal diameter is less than 50% of the repair tissue diameter; and severe, where it surpasses 50% of the repair tissue diameter.

A semiquantitative assessment of the morphological state following a cartilage repair intervention was conducted utilizing the MOCART 2.0 [6]. See the Supplementary Material for more details.

The evaluations were carried out by a highly experienced senior musculoskeletal radiologist (S.T.) with more than three decades of experience in the field. These assessments were completed 3 months, 12 months, and 60 months following the cartilage repair procedure.

To compare the morphological aspect in different kinds of BMELS, a modified MOCART score was created (more details in the Supplementary Material).

Regarding the different types of BMELS over the course of 60 months, BMELS were categorized into the following three groups:

In the first, “no edema” group, no edema-like lesion was present during the whole follow-up period subchondral to the treated area. In the second “typical edema” group, an edema-like lesion was observed but diminishing from three to 60 months. In the third “atypical edema” group, atypical progress of the edema (getting larger; new appearance after 12 months or 60 months; first getting smaller, then becoming larger; no size dynamic at all) was seen.

### Clinical outcome

To evaluate the clinical progress in this study, patient-reported measures were performed. In this study, the knee injury and osteoarthritis outcome score (KOOS) and subjective International Knee Documentation Committee (IKDC) score were used as a clinical correlation for this study. They were performed separately by each site in this multicenter study according to the general recommendations [7, 8]. More Information is in the Supplementary Material.

### Statistical evaluation

A biomedical statistician performed the statistical analysis using IBM SPSS version 24.0.1 for Windows (IBM). The images were made using GraphPad Prism version 10 (GraphPad Software, www.graphpad.com) and SPSS. Simulation-based power analysis (10,000 random iterations) was performed using R (version 4.2.1; R Core Team, 2022). A significance level of 0.05 or lower was deemed to indicate statistical significance.

Details of the statistical analysis have been moved to the Supplementary Material.

## Results

The MRI sub-study comprised a cohort of 45 individuals (13 females and 32 males with a mean age of 40.09 ± 10.25 at surgery) diagnosed with articular cartilage abnormalities (International Cartilage Repair Society III + IV). Among them, 28 patients underwent MACI treatment, while 17 patients underwent MFX treatment. The assessment of BMELS adjacent to the transplant in the subchondral bone was conducted at three-time points, as described previously. The distribution is shown in Table 2.

**Table 2:** Appearance of no BMELS, minor, and severe BMELS divided according to the treatment groups

Time	Group	No BMELS, (%)	Minor BMELS, (%)	Severe BMELS, (%)	Total
3 months	MFX	8 (47.1)	1 (5.9)	8 (47.1)	17
3 months	MACI	21 (75)	4 (14.3)	3 (10.7)	28
12 months	MFX	5 (29.4)	8 (47.1)	4 (23.5)	17
12 months	MACI	12 (42.9)	10 (35.7)	6 (21.4)	28
60 months	MFX	4 (23.5)	9 (52.9)	4 (23.5)	17
60 months	MACI	12 (42.9)	10 (35.7)	6 (21.4)	28

Age at intervention and gender were analyzed according to the pattern of BMELS (no BMELS, typical BMELS, and atypical BMELS); no significant differences in age at intervention were found between the groups (*p* = 0.596). Regarding gender differences, the distribution by sex within each BMELS class was as follows: No BMELS group had 12.5% female and 87.5% male, the typical BMELS group had 33.3% female and 66.7% male, and the atypical BMELS group had 32.1% female and 67.9% male. Chi-squared tests showed no significant associations between BMELS type and sex (*p* = 0.53).

Our results reveal no statistically significant differences in the incidence of BMELS between MFX and MACI treatment at 3 months, 12 months, and 60 months; however, a trend toward more BMELS after MFX was revealed only at the three-month follow-up (*p*-value 3 months: 0.060; 12 months 0.194; 60 months 0.372).

For the area of these BMELS, quantitative analysis of edema areas at 3 months revealed a mean area of 31.6 mm² ± 56.2, indicating substantial variability in edema sizes within this time frame (MFX: 54.6 ± 70.5; MACI: 17.6 ± 40.8). Similarly, at 12 months, the mean edema area increased to 43.5 mm² ± 62.6 (MFX: 40.0 ± 14.5; MACI: 45.8 ± 12.3). By 60 months, the mean edema area decreased to 29.5 mm² ± 35.1 (MFX: 37.8 ± 8.5; MACI: 24.5 ± 6.6).

**Fig. 2:**
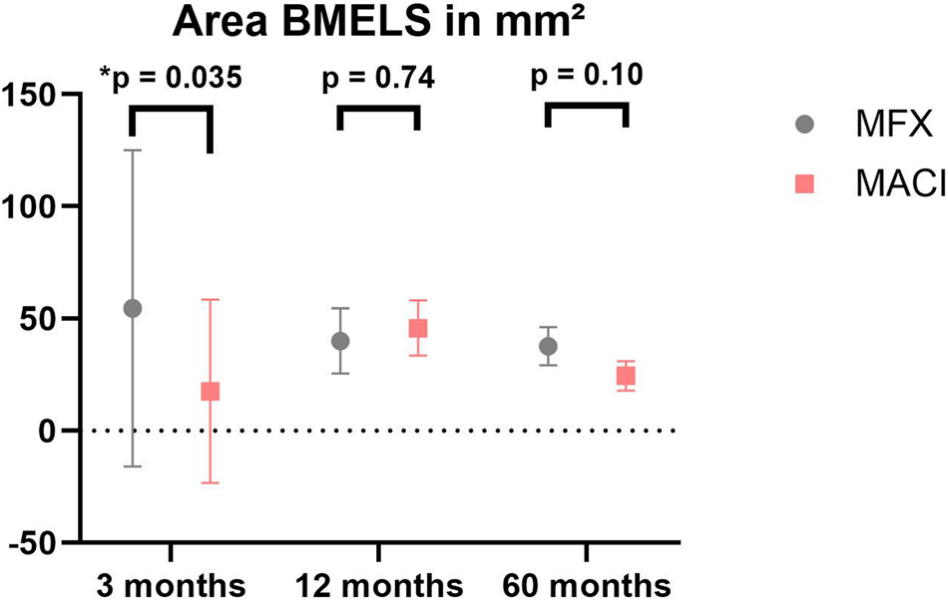
Area of BMELS in mm²; Larger BMELS were seen after 3 months in the MFX group, no differences were seen after 12 and 60 months. No significant difference between the size of the initial BMELS and edema progression patterns after 60 months was detected

Differences in edema areas among the treatment groups were analyzed. After 3 months, significant differences in edema areas between the MFX and MACI groups (*p* = 0.035) were found, indicating larger edema areas after MFX. However, at the 12-month mark, there was no significant difference in edema areas within the treatment groups (*p* = 0.736). Likewise, at the 60-month mark, no significant difference in edema areas was observed (*p* = 0.098), illustrated in Fig. 2.

Correlations were examined between the size of BMELS three and 60 months after surgery and between the IKDC score and the overall KOSS score after 60 months. No significant correlations were found between the size of BMELS at 3 months and the IKDC score and the overall KOOS score (Pearson correlation coefficient between IKDC and the size of the BMELS was −0.214 (*p* = 0.159)). Between the KOOS score and the size of the BMELS, the correlation coefficient was −0.185 (*p* = 0.222). For the size of the BMELS after 60 months, the correlation coefficient with the IKDC score was 0.165 (*p* = 0.279), and with the overall KOOS score, it was 0.109 (*p* = 0.476).

To evaluate whether the size of existing BMELS after 3 months is a factor in the differentiation between atypical and typical edema progress, we compared only cases with BMELS after 3 months and excluded all cases with no edema-like lesion after 3 months. The statistical evaluation consisted of 16 cases, with nine exhibiting a typical progress of BMELS and seven cases displaying an atypical progress over 60 months. The two-tailed asymptotic significance in this Mann–Whitney *U*-test was found to be 0.266, suggesting no statistically significant difference between the size of the initial BMELS and edema progression patterns.

**Fig. 3:**
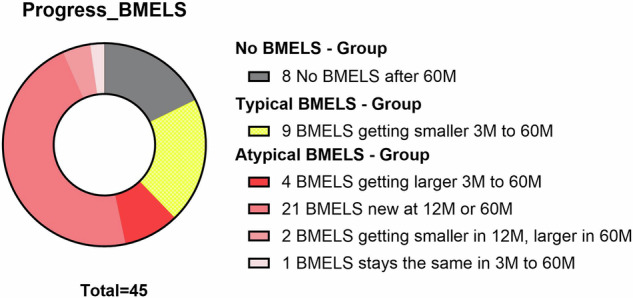
Specific progress of BMELS in the course of 60 months. We classified BMELS into three groups: no BMELS (*n* = 8). “Typical BMELS” (*n* = 9), in which BMELS were getting smaller. “Atypical BMELS” (*n* = 28), in which BMELS were getting larger or appeared for the first time at 12 M/60 M or showed no difference in size

**Fig. 4:**
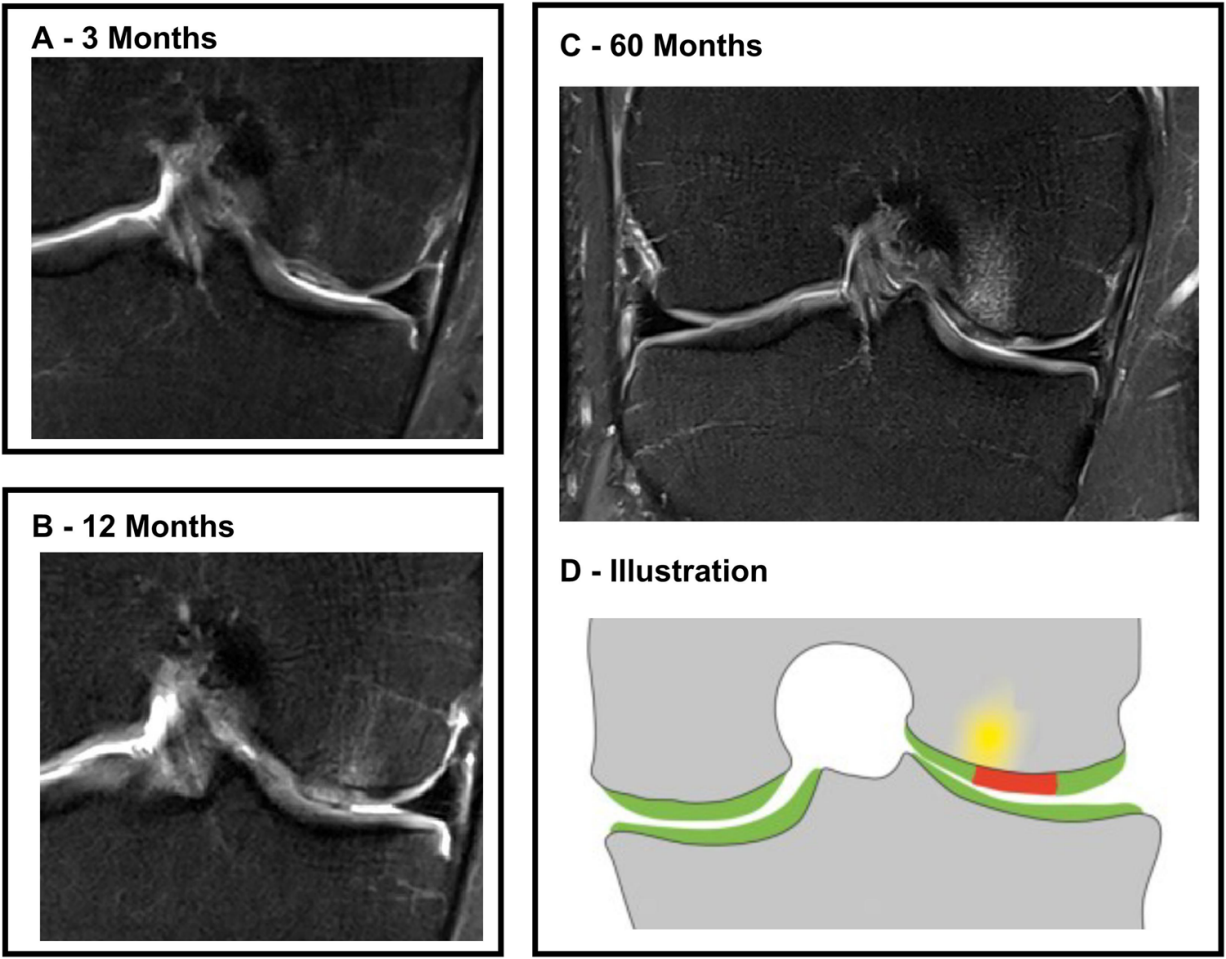
Postoperative course of an atypical BMELS: 27-year-old male patient after MACI of a focal cartilage lesion on the medial femoral condyle. **A** After 3 months: only small BMELS adjacent to the graft. **B** At 12 months and (**C**) at 60 months: BMELS becomes larger. The overall KOOS score improves from 80 (3 M), 90 (12 M) to 95 points (60 M), indicating a good clinical outcome. The modified MOCART score remains stable at 65 points (3 M) and 60 points (60 M). **D** Illustrated cartilage graft (red) and adjacent BMELS (yellow). Taken from Schreiner et al [6]

Figure 3 depicts the patterns of progression observed within the lesions and classifies the lesions based on specific characteristics. Figure 4 shows a clinical example of an atypical course of BMELS in a 27-year-old patient with an otherwise good clinical outcome.

The progression and characteristics of BMELS over a 60-month period with regard to the different treatments were also evaluated, and illustrated in the Supplementary Material. Our analysis focused on different types of progression and specific features exhibited by these lesions. Within the MACI treatment group, comprising 28 cases, six patients showed no BMELS, nine individuals exhibited a trend of BMELS getting smaller from three to 60 months (typical progress), and 19 patients displayed an atypical progress. Conversely, in the MFX treatment group, consisting of 17 cases; two patients showed no BMELS, eight individuals showed a typical progress, and nine patients showed an “atypical progress”.

In the MFX group, 12% showed no BMELS, 35% exhibited typical progress, and 53% displayed atypical progress. For MACI, 21% showed no BMELS, 11% demonstrated typical progress, and 68% exhibited atypical progress.

The analysis of typical and atypical BMELS progress in MFX/MACI after 60 months was conducted using Fisher’s exact test to assess statistical significance. The obtained *p*-value was 0.1176, indicating a lack of statistical significance with a two-sided test. Nevertheless, a tendency toward more atypical progress of BMELS after 60 months was observed in the MACI group.

### T2 mapping

There were no significant differences in the T2 ratio of the treated area at 3 months, 12 months, and 60 months in patients with or without BMELS at the respective time points (*p*-value 3 months = 0.943, 12 months = 0.206, and 60 months = 1.00). In addition, no significant differences were seen in the size of edema-like lesions (“no BMELS,” “minor BMELS,” and “severe BMELS”) at any time point and the T2 ratio after 60 months (quantification of BMELS after 3 months (*p* = 0.168), after 12 months (*p* = 0.886), and after 60 months (*p* = 0.724)).

The differences in the T2 ratio after 60 months and the progression of BMELS were meticulously examined between the “no edema,” “typical edema,” and “atypical edema” groups. The corresponding asymptotic significance *p*-value was calculated at 0.600. The results indicated no statistically significant differences in the edema progression patterns among the three specified groups and the T2 ratio.

Furthermore, no significant differences were found in the independent treatment groups between “no edema,” “typical edema,” and “atypical edema” groups regarding the T2 ratio of the treated area after 3 months (Kruskal–Wallis *p*-value: MFX = 0.58, MACI = 0.42) and after 60 months (Kruskal–Wallis *p*-value: MFX = 0.661, MACI = 0.649).

The average values of the T2 ratio of the treatment groups are shown in Table 3.

**Table 3:** Mean T2 ratio of the treatment group

Time	Mean T2 ratio MFX	Mean T2 ratio MACI	*p*-value
3 months	1.3587 (SD: 0.34)	1.2876 (SD: 0.31)	0.64
12 months	1.1959 (SD: 0.31)	1.0555 (SD: 0.22)	0.175
60 months	1.1231 (SD: 0.24)	1.0456 (SD: 0.27)	0.198

No significant differences were seen between MFX and MACI

### MOCART score 2.0

The MOCART score of 2.0 was evaluated after 3 months, 12 months, and 60 months. In one patient, the evaluation was not possible because of artifacts after ACL reconstruction in the area of the transplant. Other reasons for focal BMELS adjacent to the treated area were excluded through MRI, like insufficient fractures, new cartilage lesions, or delamination of cartilage. The MOCART 2.0 score after 3 months exhibited a mean total score of 65.7 ± 19.9. After 12 months, the mean MOCART score was 74.1 ± 20.4, and after 60 months the mean total score was 76.6 ± 13.0.

**Fig. 5:**
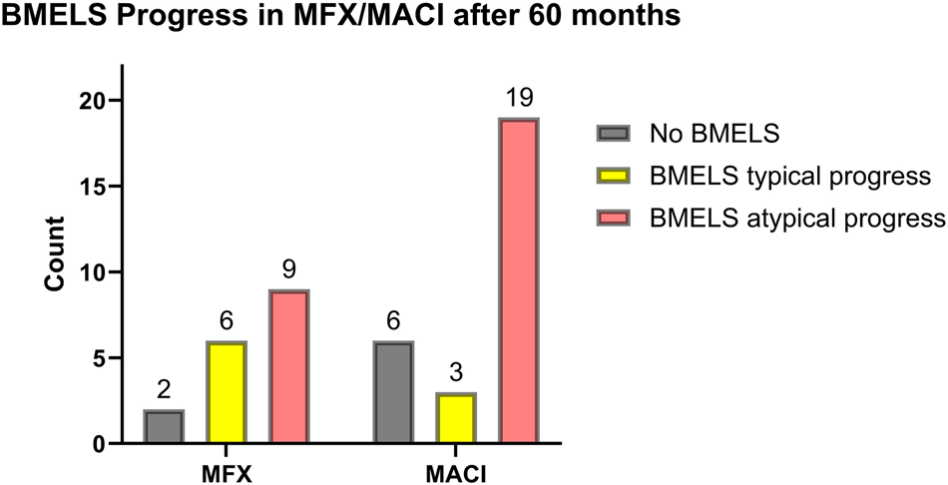
Modified MOCART 2.0 score in atypical, typical, and no BMELS. No significant differences were observed

To obtain statistical validity regarding the morphology of the treated area using the MOCART 2.0 score, a modified MOCART score was built without the subcategory “subchondral changes,” as previously mentioned. This modified MOCART score was compared between the groups “no BMELS” (*n* = 8, mean = 65.6), “typical progress of BMELS” (*n* = 9, mean = 63.9), and “atypical progress of BMELS” (*n* = 27, mean = 59.6). No significant difference in the ranks of the three groups (*p* = 0.186) was observed (Fig. 5).

### Clinical scores

The results of the KOOS and IKDC scores were evaluated before surgery and 3 months, 12 months, and 60 months after surgery. Compared to before surgery, all scores improved. The IKDC score increased by an average of 36.3 ± 23.7 points, and the overall KOOS score increased by 32.4 ± 23.5 points.

**Fig. 6:**
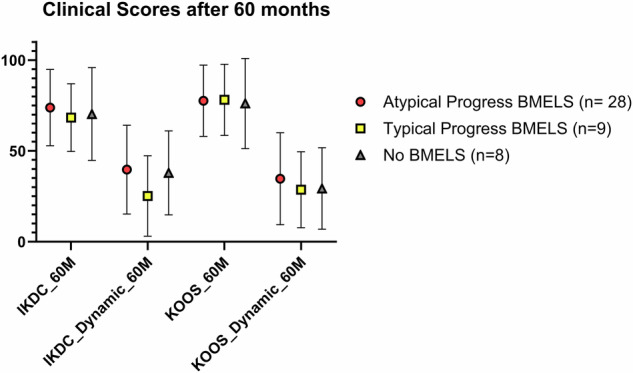
Clinical scores; IKDC score and overall KOOS score after 60 months. Dynamic of IKDC score and overall KOOS score in comparison between baseline and 60 months. No significant differences were seen

The data were categorized based on the type of BMELS (“no BMELS,” “atypical BMELS,” and “typical BMELS”). As mentioned before, we used the dynamic of KOOS and IKDC scores from baseline and after 60 months, as well as the score values after 3 months, 12 months, and 60 months. The results are shown in Table 4 and Fig. 6. No significant differences were seen between the groups. Particular attention was paid to differences in pain between different BMELS types: There were no statistically significant differences in the Pain KOOS subscore at 3 months (*p* = 0.125), 12 months (*p* = 0.519), or 60 months (*p* = 0.361).

**Table 4:** Clinical scores of “typical,” “atypical,” and “no BMELS” were compared

Scores	No BMELS	Typical BMELS	Atypical BMELS	*p*-value
	Mean (SD)	Mean (SD)	Mean (SD)	
Dynamic IKDC	37.9; (23.1)	25.1; (22.1)	39.7; (24.4)	0.277
Dynamic pain—KOOS	19.8; (19.5)	21.9; (17.6)	31.8; (18.8)	0.205
Dynamic symptoms—KOOS	19.1; (19.9)	23.0; (26.1)	26.5; (23.4)	0.635
Dynamic function daily—KOOS	24.2; (22.8)	19.2; (14.7)	31.1; (23.8)	0.209
Dynamic sport—KOOS	40.7: (23.2)	27.2; (28.7)	44.5; (33.3)	0.347
Dynamic quality of life—KOOS	43.8; (34.2)	35.4; (26.9)	35.5; (30.8)	0.886
Dynamic overall KOOS score	29.3; (22.4)	28.6; (20.9)	34.7; (25.4)	0.568
KOOS 3 months	49.3; (11.9)	67.4; (13.5)	59.5; (15.3)	0.052
KOOS 12 months	67.1; (17.0)	77.9; (12.6)	72.5; (16.1)	0.475
KOOS 60 months	77.2; (24.7)	78.1; (19.6)	78.4; (19.6)	0.983
IKDC 3 months	45.5; (13.2)	58.9; (11.9)	51.2; (15.0)	0.165
IKDC 12 months	54.7; (16.4)	73.4; (15.7)	65.5; (20.8)	0.152
IKDC 60 months	72.4; (24.4)	68.3; (18.6)	74.7; (20.8)	0.744

No significant differences were seen in any of the scores

### Simulation-based power analysis and interrater agreement

Regarding the power of the statistical tests, we performed a simulation-based power analysis (power 10,000 random iteration) for our adapted MOCART 2.0 score, assuming normal distributions; a mean difference of 15 in one of the groups (which is a criterion for clinically significant difference, like shown in other studies [9]) and an SD of 14 (which was the highest SD observed among the three groups). This analysis showed a power of 0.83.

Assuming a significant difference of 20 points regarding clinical scores (IKDC and KOOS) and an SD of 24 (which was the highest SD observed among the three groups), our simulation-based power analysis demonstrated a power of 0.60. Details are shown in Table 5.

**Table 5:** Simulation-based power analysis for our adapted MOCART 2.0 score, IKDC, and KOOS score after 60 months

	No BMELS	Typical BMELS	Atypical BMELS	Difference (in means) considered clinically significant	SD assumed for simulation	Power 10,000 random iteration
MOCART 60 M (mean ± SD)	65.6 ± 8.2	63.9 ± 13.6	59.6 ± 12.6	15	14	0.83
IKDC 60 M (mean ± SD)	72.4 ± 24.4	68.3 ± 18.6	74.7 ± 20.8	20	24	0.6
KOOS 60 M (mean ± SD)	77.2 ± 23.2	78.1 ± 19.6	78.4 ± 19.6	20	24	0.6

Perfect interrater agreement (kappa = 1) was found for the presence of BMELS at 3 months, 12 months, and 60 months and for a specific type of BMELS. Intraclass correlation was excellent (ICC > 0.99) for the size of BEMLS at 3 months, 12 months, and 60 months.

## Discussion

BMELS after cartilage repair was a common finding in our cohort. No evidence was found that the appearance, dimensions, or dynamics of size influenced clinical scores, morphological MRI, or biochemical properties of the treated area at 60 months. Numerous studies have highlighted the important connection between subchondral edema and the articular cartilage above it [10]. Nevertheless, the significance of subchondral bone lesions and persistent bone marrow edema after cartilage repair procedures, such as MACI and MFX, remains uncertain and is a topic of extensive debate, as shown in a meeting of experts in the field in 2015, where a level of disagreement was reported regarding the management of BMELS [11].

In this study, we demonstrated that dimensional changes of BMELS as well as the specific course (typical, atypical, or no BEMLES) following MACI and MFX procedures do not significantly affect clinical scores, morphological MRI outcomes, or biochemical characteristics of the treated region over a 60-month period in a comparatively large cohort of 45 patients.

The appearance of BMELS are common radiological finding after cartilage repair. Incidences are very heterogeneous: Between 12% after five years after autologous chondrocyte implantation (ACI) [12] or 47% after 60 months undergoing MACI [13]. In our study, 57% of patients after MACI showed BMELS. Regarding the huge differences in the frequency of BMELS, the different forms of ACI or MACI must be considered. Results from Niethammer et al, who also used the same MACI technique with a comparable defect size (mean 5.3 cm²) showed comparable results after 12 months (no BMELS in 45%, in our study: 42% after 12 months) and in the long term (36 months with no BMELS in 37%; In our study 43% after 60 months) [14]. The heterogeneous group of MACI procedures must be considered, and the appearance of the BMELS can obviously vary depending on the generation and specific form of the MACI treatment.

Subchondral edema is frequently found in the short term after MFX, and, during normal maturation, it should decrease in size in later follow-ups [15]. Alparslan and colleagues reported, in 2001, that the altered subchondral signal area typically decreases but rarely disappears entirely [16].

It is fundamental to distinguish between the short-term appearance of BMELS postoperatively and their appearance in the mid- and long-term, with a new occurrence or an increase in the size of the lesions in the later course.

After MFX, the early postoperative appearance of BMELS has been described [16]. In our study, there were no significant differences in the incidence of BMELS in MACI and MFX, but the size of BMELS after MFX was significantly larger after 3 months compared to those after MACI, which also balanced out after 12 months in the treatment groups. This result is not surprising considering the invasiveness of this procedure as it relates to the penetration of the subchondral bone.

We found no correlation between BMELS size and clinical scores after 60 months. These results seem to characterize these postoperative BMELS in a different way than comparable edema-like lesions, which appear without surgery as a prognostic factor for OA [17]. Thus, it is of tremendous importance to recognize and treat BMELS after cartilage repair differently than non-specific edema-like lesions in OA. BMELS are non-specific with a variety of different histologies. Zanetti et al correlated histology and MR imaging of BMELS in osteoarthritic knees and reported various non-specific histological abnormalities and that edema is not a major component of the MR imaging signal intensity abnormalities [18].

Nevertheless, we could show that, in both treatment groups, different courses of BMELS do not affect any clinical scores or morphological MRI results (proved with the MOCART 2.0 score) or biochemical properties (proved with T2 mapping). Several studies have shown similar results [14, 19], but, to our knowledge, we are the first to combine clinical scores, morphologic results, and biochemical properties and focus on one of the most discussed and one of the most common “complications” After cartilage repair. Some studies describe subchondral edemas as a potential prognostic factor in the initial stages after ACI, meaning that no BMELS in the short term is a negative prognostic factor [20]. However, our study did not reveal any correlations or significant differences in the appearance or absence of BMELS in our treatment groups.

Some authors believe that the delayed or sporadic appearance of BMELS after 12 months might indicate inadequate biomechanical properties in the MACI cartilage. The local orientation of the collagen fibers and the amount of mobile water are reflected in the T2 mapping of the articular cartilage [21]. T2 mapping allows the visualization of the maturation of the graft [22]; the smaller the difference in T2 values between the repair tissue and native cartilage after maturation, the better the clinical outcome [23]. We could demonstrate that there were no significant differences in the T2 ratio over 60 months after MACI or MFX in patients without BMELS, with “typical” or “atypical” BMELS No significant differences were seen in the T2 ratio of the treated area after 3 months in MFX and MACI, either, revealing no predictive value of T2 mapping to predict BMELS in the long term.

Furthermore, our findings demonstrate that the extent of edema does not influence the outcome after 60 months and does not influence whether the course of edema is typical or atypical. Based on our results, the most clinically relevant time point for assessing cartilage repair procedures is approximately 12 months post-surgery. Several MRI findings, including hyperintense repair tissue signal, subchondral edema, and effusion, are part of the normal repair process, but are classified as abnormal MRI findings. Consequently, existing MRI classification systems and many of their individual parameters may not offer significant insights in the early stages after surgery. However, between s6- and 60 months post-surgery, MRI evaluations may prove valuable for assessing cartilage repair tissue in patients who continue to experience persistent symptoms, which has already been described by several studies [13, 20].

It is important to consider the timepoint of the appearance of BMELS, because in MACI (and also in MFX), the maturation process seems to be complete after two years [13]. Consequently, the occurrence of BMELS after two years cannot be explained by a physiological healing process. Genovese et al found BMELS in 20% of cases after 30 months and in 47% of cases after 60 months post-surgery; A significant finding was that BMELS at the 30-month mark did not show improvement until the 60-month period, and that these newly emerging BMELS after 30 months showed no correlations with clinical scores and the modified MOCART score [13]. One limitation of our study is that we did not include a 24-month follow-up after the completed maturation of the treated area. In our cohort, there were several BMELS that disappeared or newly appeared at the 60-month follow-up compared to the 12-month follow-up, which could still be explained as a prolonged healing process. More studies are needed that compare and correlate the new appearance of BMELS after two years regarding clinical scores.

Other limitations of this study include that this multicenter study involved the use of various MRI systems, despite employing predefined MRI protocols. Moreover, some patients had to be excluded due to non-participation in follow-up investigations and the absence of certain MRI sequences. Two groups (no BMELS (*n* = 8) and typical BMELS (*n* = 9)) are quite small although simulation-based power analysis has proven a good power of the used statistical test. However, the number of patients examined at a 5-year follow-up on cartilage repair is comparable to other studies and we are confident, that we have taken stepstones in the clinical evaluation of BMELS after cartilage repair. There is heterogeneity in the histology of BMELS. We have excluded reasons other than cartilage repair for new BMELS adjacent to the treated area, such as insufficient fractures, new cartilage lesions, or delamination. However, other reasons for the new BMELS cannot be excluded with certainty. Finally, it should be mentioned, that the utilization of the adjusted MOCART score in this study poses challenges in terms of comparing findings to those of other studies.

## Conclusion

BMELS frequently appear after cartilage repair. Based on our data, no evidence can be found that the appearance, dimension, or size dynamics of the BMELS after MACI and MFX have an influence on the clinical score, morphologic MRI results, or biochemical properties of the treated area after 60 months.

## Supplementary information

ELECTRONIC SUPPLEMENTARY MATERIAL

## Abbreviation

ACI: Autologous chondrocyte implantation
BMELS: Bone marrow edema-like signal
IKDC: International Knee Documentation Committee
KOOS: Knee injury and osteoarthritis outcome score
MACI: Matrix-induced autologous chondrocyte implantation
MFX: Microfracturing
MOCART 2.0: Magnetic resonance observation of cartilage repair tissue
PD: Proton density
TSE: Turbo spin echo
