# Supplementary material for: Bone marrow edema-like signal after cartilage repair does not affect outcomes in a five-year follow-up
Source: Eur Radiol. 2024 Sep 16;35(4):1808–17. doi: 10.1007/s00330-024-11078-8 (PMC11913985; doi:10.1007/s00330-024-11078-8)
Supplement: Supplementary file 1 — ELECTRONIC SUPPLEMENTARY MATERIAL [file 330_2024_11078_MOESM1_ESM.pdf]

Bone marrow edema-like signal after cartilage repair does not affect outcomes in a five-year follow-up

ELECTRONIC SUPPLEMENTARY MATERIAL

Figure: “Progress of BMELS in MFX and MACI”

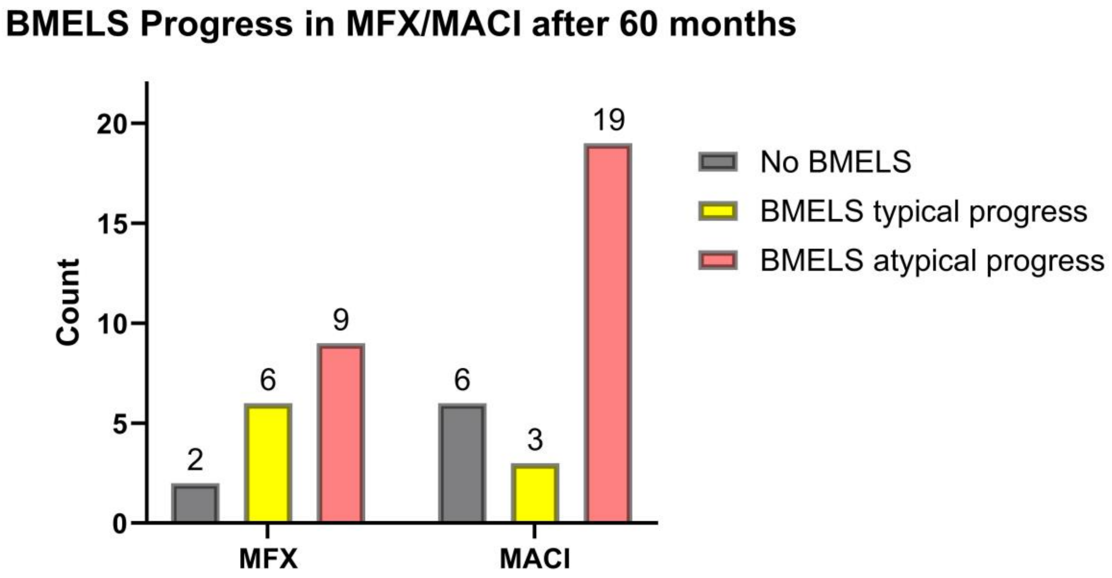

Progress of BMELS in MFX and MACI over the course of 60 months. No significant differences were found between atypical and typical BMELS between the treatment groups, but a trend toward more atypical BMELS in MACI was noted.

Extended Methodology:

Inclusion and Exclusion Criteria

The enrollment criteria required that patients had a localized articular cartilage defect specifically in the femoral condyle or the trochlea of the knee. These defects were required to be classified as grade III or IV according to the International Cartilage Repair Society (ICRS) classification, with a defect size ranging from 2 to 6 cm^2. The patient has an intact meniscus, with up to 50% resection permitted. If no data is available from the medical history, the status of the meniscus can be estimated at Visit 1. The patient has a stable knee joint or adequately reconstructed ligaments; if not, ligament repair must be performed before, during, or within six weeks after cartilage treatment). The patient has full range of motion in the affected knee joint or a loss of no more than 10° in extension and flexion. The exclusion criteria encompassed the inability to undergo specific medical imaging, prior cartilage repair surgeries, degenerative joint diseases (Kellgren and Lawrence grade of 2 or higher), inflammatory arthritis, malalignment (specifically valgus- or varus deformity), systemic diseases, infections, a body mass index (BMI) exceeding 35 kg/m^2, substance abuse, cognitive impairments, has an active systemic or local microbial infection at the site of surgery or known history of cancer within the past five years.

MR Examination

All MRI examinations were performed on a 3 Tesla whole-body MR scanner: MRI scans were performed on Siemens 3T MRIs (Siemens Healthineers, Erlangen, Germany), Philips Medical Systems 3T MRIs (Philips Medical Systems, Best, Netherlands), and a GE Medical Systems 3T MRI (GE Medical Systems, Chicago, IL, USA). Vender dedicated multielement knee coils (Eight to 16 coils) were used for this multicenter study.

The MR imaging protocol consisted of two main components: a morphological component and a compositional component with T2 mapping. The morphological component included the Turbo Spin Echo (TSE), Proton Density (PD), TSE T2 weighted (w), and SE T1w sequences. The T2 mapping component included a multi-echo multi-slice sequence. The detailed presentation of the comprehensive magnetic resonance

(MR) examination methodology and its accompanying sequence parameters are listed in Table 1 (already mentioned in other studies of our research group) [1].
The central reading facility received all images and conducted T2 mapping using a two-parameter exponential fitting technique. In addition, they conducted segmentation and grading for cartilage restoration.

Mocart Score

A semiquantitative assessment of the morphological state subsequent to a cartilage repair intervention was conducted utilizing the MOCART 2.0 [2].
This was done to methodically record and document the condition of the cartilage repair site and the adjacent tissues by using a point-scale evaluation. The demonstrated reliability, reproducibility, and application of this technique across various surgical cartilage restoration methods have been well-established in the literature [3, 4].
The scoring system comprises seven sub-scores, with each component contributing to a maximum score of 100, that evaluate different aspects of cartilage repair. These sub-scores include the volume fill of the cartilage defect, integration into the adjacent cartilage, surface characteristics of the repair tissue, structural properties of the repair tissue, signal intensity of the repair tissue, presence of bony defect or bony overgrowth, and subchondral changes. Each sub-score is assigned a range of points to quantify its respective evaluation.

Modified Mocart Score

To compare the morphological aspect in different kinds of BMELS, aA modified MOCART score was created. Subchondral changes are weighted with up to 20 points in the original score. In order to show only the influence of BMELS on the other morphological MRI aspects, the subcategory 'subchondral changes' was removed. After scoring, only the values of the above-mentioned subcategories were added, excluding "subchondral changes."

Clinical Outcome

The KOOS is a self-reported questionnaire that assesses five subscales of knee function: pain; symptoms; daily living function; sport and recreation function; and quality of life. The IKDC is a clinician-administered questionnaire that assesses overall knee function. These scores were employed as per standard guidelines. Scores evaluated at baseline before the intervention and three, 12, and 60 months after intervention were included in this study.

To demonstrate the dynamic of IKDC and KOOS scores, we subtracted the baseline value from the corresponding score after 60 months to represent the clinical improvement after the intervention.

Statistical Evaluation

A biomedical statistician performed the statistical analysis using IBM SPSS version 24.0.1 for Windows (IBM, Chicago, IL). The representation of data metrics typically involves the mean value accompanied by the standard deviation (SD). The images were made using GraphPad Prism version 10 (GraphPad Software, La Jolla, California, USA, www.graphpad.com) and SPSS.

Simulation-based power analysis (10000 random iterations) was performed using R (Version 4.2.1; R Core Team, 2022).

Interrater agreement was determined by calculating the percentage of agreement and Cohen's Kappa for categorical variables (Presence of BMELS at 3, 12 and 60 months as well as for specific kind of BMELS), and intraclass correlation coefficients (ICC) (two-way mixed-effects model, absolute agreement) for numerical variables (Size of BEMLS after 3, 12 and 60 months).

Prior to conducting the Mann-Whitney U-test and Wilcoxon rank test, we performed a normality test to assess whether the data was normally distributed. We used the Shapiro-Wilk test, which is a non-parametric test for normality. The results of the Shapiro-Wilk test showed that the data was not normally distributed ($p < 0.05$). A significance level of 0.05 or lower was deemed to indicate statistical significance.

References Supplementary Material

1. Juras V, Szomolanyi P, Janáčová V, et al (2021) Differentiation of Cartilage Repair Techniques Using Texture Analysis from T2 Maps. Cartilage 13:718S-728S. https://doi.org/10.1177/19476035211029698

2. Schreiner MM, Raudner M, Marlovits S, et al (2021) The MOCART (Magnetic Resonance Observation of Cartilage Repair Tissue) 2.0 Knee Score and Atlas. Cartilage 13:571S-587S. https://doi.org/10.1177/1947603519865308

3. Schreiner MM, Raudner M, Röhrich S, et al (2021) Reliability of the MOCART (Magnetic Resonance Observation of Cartilage Repair Tissue) 2.0 knee score for different cartilage repair techniques-a retrospective observational study. Eur Radiol 31:5734–5745. https://doi.org/10.1007/S00330-021-07688-1

4. Jung M, Karampinos DC, Holwein C, et al (2021) Quantitative 3-T Magnetic Resonance Imaging After Matrix-Associated Autologous Chondrocyte Implantation With Autologous Bone Grafting of the Knee: The Importance of Subchondral Bone Parameters. Am J Sports Med 49:476–486. https://doi.org/10.1177/0363546520980134
